# Supplementary material for: Remarkable Nonlinear Properties of a Novel Quinolidone Derivative: Joint Synthesis and Molecular Modeling
Source: Molecules. 2022 Apr 7;27(8):2379. doi: 10.3390/molecules27082379 (PMC9028933; doi:10.3390/molecules27082379)
Supplement: Supplementary file 1 [file molecules-27-02379-s001.zip › molecules-1656232-supplementary.pdf]

Support Information

Remarkable Nonlinear Properties of a Novel Quinolidone Derivative: Joint Synthesis and Molecular Modeling

Clodoaldo Valverde[1,2,*], Rafael S. Vinhal[3], Luiz F.N. Naves[1], Jean M.F. Custódio[4], Basílio Baseia[3,5], Heibbe Cristhian B. de Oliveira[6], Caridad N. Perez[6], Hamilton B. Napolitano[1], and Francisco A. P. Osório[3,7]

[1] Universidade Estadual de Goiás, 75001-970, Anápolis, GO, Brazil.
[2] Universidade Paulista – UNIP, 74845-090, Goiânia, GO, Brazil.
[3] Instituto de Física, Universidade Federal de Goiás, 74.690-900, Goiânia, GO, Brazil.
[4] University of Notre Dame, 46656, Notre Dame, IN, USA.
[5] Departamento de Física, Universidade Federal da Paraíba, 58051-970, João Pessoa, PB, Brazil.
[6] Instituto de Química, Universidade Federal de Goiás, 74.690-900, Goiânia, GO, Brazil.
[7] Escola Politécnica, Pontifícia Universidade Católica de Goiás, 74605-100, Goiânia, GO, Brazil.
* Correspondence: valverde@ueg.br

Abstract: A novel 4(1H) quinolinone derivative (QBCP) was synthesized and characterized with single crystal X-ray diffraction. Hirshfeld surfaces (HS) analyses were employed as a complementary tool, to evaluate the crystal intermolecular interactions. The molecular global reactivity parameters of QBCP were studied using HOMO and LUMO energies. In addition the molecular electrostatic potential (MEP) and the UV-Vis absorption and emission spectra were obtained and analyzed. The supermolecule (SM) approach was employed to build a bulk with 474,552 atoms to simulate the crystalline environment polarization effect on the asymmetric unit of the compound. The nonlinear optical properties were investigated using the density functional method (DFT/CAM-B3LYP) with the Pople's 6-311++G(d,p) basis set. The quantum DFT results of the linear polarizability, the average second-order hyperpolarizability and the third-order nonlinear susceptibility values were computed and analyzed. The results show that the organic compound (QBCP) has great potential for application as a third-order nonlinear optical material.

LIST OF TABLES

Table S1. DFT / CAM-B3LYP / 6-311 ++ G (d,p) Coordinates of asymmetric unit A QBCP. 3
Table S2. DFT / CAM-B3LYP / 6-311 ++ G (d,p) Coordinates of asymmetric unit B QBCP... ... 5
Table S3. DFT / CAM-B3LYP / 6-311 ++ G (d,p) Coordinates of asymmetric unit C QBCP. ... 错误!未定义书签。
Table S4. DFT / CAM-B3LYP / 6-311 ++ G (d,p) Coordinates of asymmetric unit D QBCP. 9
Table S5. DFT / CAM-B3LYP / 6-311 ++ G (d,p) charges ChelpG of asymmetric unit A QBCP....11
Table S6. DFT / CAM-B3LYP / 6-311 ++ G (d,p) charges ChelpG of asymmetric unit B QBCP.13
Table S7. DFT / CAM-B3LYP / 6-311 ++ G (d,p) charges ChelpG of asymmetric unit C QBCP.15
Table S8. DFT / CAM-B3LYP / 6-311 ++ G (d,p) charges ChelpG of asymmetric unit D QBCP.17
Table S9. DFT / CAM-B3LYP / 6-311 ++ G (d,p) Dipole moment of asymmetric unit A QBCP.19
Table S10. DFT / CAM-B3LYP / 6-311 ++ G (d,p) Dipole moment of asymmetric unit B QBCP.20
Table S11. DFT / CAM-B3LYP / 6-311 ++ G (d,p) Dipole moment of asymmetric unit C QBCP.21
Table S12. DFT / CAM-B3LYP / 6-311 ++ G (d,p) Dipole moment of asymmetric unit D QBCP.22
Table S 13. DFT / CAM-B3LYP / 6-311 ++ G (d,p) *IDRI* Average Second Hyperpolarizability (esu) ………………………………………………………………………………………………... 23

Table S14. DFT / CAM-B3LYP / 6-311 ++ G (d,p) average linear polarizability………………....24
Table S15. DFT / CAM-B3LYP / 6-311 ++ G (d,p) the dynamic third-order nonlinear susceptibility……………………………………………………………………………….......25
Table S16. DFT / CAM-B3LYP / 6-311 ++ G (d,p) the refractive index, Clausius-Mossotti Relation……………………………………………………………………………….......26

LIST OF FIGURES

Figure S1. The unit cell with the four asymmetric units A,B,C, and D………………………… 27

Figure S2. Dipole Moment of the four asymmetric units A,B,C, and D……………………….. 28

Figure S3. The d_{norm} property of Hirshfeld surfaces interactions presenting red spots between atoms. Fingerprints show the frequency of the type of interactions in the structure. The shape index shows by the red concave regions, a characteristic of aromatic ring involved in atom$\cdots\pi$ interactions.

Figure S4. ORTEP map of the QBCP asymmetric unit with 50% probability. Disordered atoms have a suffix A……………………………………………………………………………… 29

Table S1. DFT / CAM-B3LYP / 6-311 ++ G (d,p) Coordinates of asymmetric unit A QBCP.

		Coordinates		
Atom	Label	x	y	z
C	C26	3.611025	7.491398	5.614547
H	H26	3.368886	8.387193	5.676937
C	C27	4.651666	7.098030	5.936108
H	H27	5.155880	7.819402	6.236732
S	S1	4.957502	3.181627	5.233981
Cl	CL1	8.389842	0.122072	-1.494116
N	N1	5.943850	3.205393	3.896034
O	O1	6.356634	6.818339	2.100726
C	C10	5.289782	3.329596	2.563851
H	H10	4.399835	2.923179	2.620056
C	C18	6.331324	3.022279	0.273830
H	H18	6.002989	3.860323	0.039770
C	C17	6.112105	2.540444	1.548399
C	C7	6.298515	5.656696	2.480304
C	C1	7.406937	5.056271	3.258603
C	C8	5.121100	4.795158	2.205046
C	C2	7.205862	3.884823	3.993655
C	C23	4.320223	4.808420	5.405029
O	O2	3.892968	2.298017	4.910809
C	C6	8.648608	5.685849	3.304540
H	H6	8.799056	6.451969	2.799266
C	C3	8.228765	3.375114	4.776511
H	H3	8.101573	2.595210	5.266887
C	C5	9.651281	5.178283	4.093967
H	H5	10.474578	5.608821	4.135365
O	O3	5.798760	2.916453	6.349837
C	C9	4.011195	5.369213	1.734674
H	H9	4.060786	6.290572	1.618824
C	C4	9.436677	4.036451	4.820545
H	H4	10.120376	3.701022	5.354351
C	C19	7.028952	2.282991	-0.659631
H	H19	7.173608	2.620349	-1.514120
C	C21	7.302413	0.535475	0.945357
H	H21	7.627325	-0.306903	1.168382
C	C22	6.611027	1.285610	1.874114
H	H22	6.477184	0.946629	2.729720
C	C11	2.725933	4.763601	1.373599
C	C20	7.505123	1.048417	-0.313527
C	C12	1.558318	5.484606	1.631983
H	H12	1.613392	6.316149	2.044741
C	C16	2.626515	3.531230	0.744075
H	H16	3.397277	3.043179	0.563254
C	C15	1.384613	3.017886	0.380328
H	H15	1.400762	2.176928	-0.016453
C	C24	3.045082	5.090587	4.985590
H	H24	2.572753	4.357291	4.662931
C	C28	5.136336	5.800852	5.892257

H	H28	5.996670	5.605695	6.186604
C	C13	0.321480	4.976330	1.281175
H	H13	-0.472522	5.425269	1.462143
C	C25	2.783081	6.543029	5.143369
H	H25	1.938824	6.833851	4.883247
Br	BR1	-1.574167	3.341603	0.349398
C	C14	0.178961	3.997504	0.804818

Table S2. DFT / CAM-B3LYP / 6-311 ++ G (d,p) Coordinates of asymmetric unit B QBCP.

		Coordinates		
Atom	Label	x	y	z
C	C26	0.276973	13.412048	3.536313
H	H26	0.519112	14.307843	3.473923
C	C27	-0.763668	13.018680	3.214752
H	H27	-1.267883	13.740052	2.914128
S	S1	-1.069505	9.102277	3.916879
Cl	CL1	-4.501845	6.042722	10.644976
N	N1	-2.055852	9.126043	5.254827
O	O1	-2.468637	12.738989	7.050134
C	C10	-1.401785	9.250246	6.587009
H	H10	-0.511837	8.843829	6.530804
C	C18	-2.443327	8.942929	8.877030
H	H18	-2.114992	9.780973	9.111091
C	C17	-2.224108	8.461094	7.602461
C	C7	-2.410518	11.577346	6.670556
C	C1	-3.518940	10.976921	5.892257
C	C8	-1.233102	10.715808	6.945814
C	C2	-3.317865	9.805473	5.157205
C	C23	-0.432225	10.729070	3.745831
O	O2	-0.004971	8.218667	4.240051
C	C6	-4.760610	11.606499	5.846320
H	H6	-4.911058	12.372619	6.351594
C	C3	-4.340768	9.295764	4.374349
H	H3	-4.213575	8.515860	3.883973
C	C5	-5.763284	11.098933	5.056893
H	H5	-6.586581	11.529471	5.015495
O	O3	-1.910763	8.837103	2.801023
C	C9	-0.123198	11.289863	7.416187
H	H9	-0.172789	12.211222	7.532036
C	C4	-5.548680	9.957101	4.330315
H	H4	-6.232379	9.621672	3.796509
C	C19	-3.140955	8.203641	9.810491
H	H19	-3.285611	8.540999	10.664980
C	C21	-3.414416	6.456125	8.205503
H	H21	-3.739328	5.613747	7.982478
C	C22	-2.723030	7.206260	7.276746
H	H22	-2.589187	6.867279	6.421140
C	C11	1.162064	10.684251	7.777261
C	C20	-3.617126	6.969067	9.464387
C	C12	2.329679	11.405256	7.518878
H	H12	2.274606	12.236799	7.106119
C	C16	1.261482	9.451880	8.406785
H	H16	0.490720	8.963829	8.587606
C	C15	2.503384	8.938536	8.770532
H	H15	2.487236	8.097578	9.167313
C	C24	0.842916	11.011237	4.165270
H	H24	1.315244	10.277941	4.487930
C	C28	-1.248339	11.721502	3.258603

H	H28	-2.108673	11.526345	2.964256
C	C13	3.566517	10.896980	7.869685
H	H13	4.360520	11.345919	7.688717
C	C25	1.104916	12.463679	4.007491
H	H25	1.949173	12.754501	4.267613
Br	BR1	5.462164	9.262253	8.801462
C	C14	3.709037	9.918154	8.346042

Table S3. DFT / CAM-B3LYP / 6-311 ++ G (d,p) Coordinates of asymmetric unit C QBCP.

Atom	Label	Coordinates		
		x	y	z
C	C26	4.164970	4.349902	12.687174
H	H26	4.407109	3.454107	12.624783
C	C27	3.124329	4.743270	12.365612
H	H27	2.620114	4.021898	12.064988
S	S1	2.818493	8.659673	13.067740
Cl	CL1	-0.613848	11.719228	19.795836
N	N1	1.832145	8.635907	14.405687
O	O1	1.419360	5.022961	16.200994
C	C10	2.486212	8.511704	15.737869
H	H10	3.376160	8.918121	15.681664
C	C18	1.444670	8.819021	18.027890
H	H18	1.773005	7.980977	18.261951
C	C17	1.663890	9.300856	16.753322
C	C7	1.477479	6.184604	15.821416
C	C1	0.369057	6.785029	15.043117
C	C8	2.654895	7.046142	16.096674
C	C2	0.570133	7.956477	14.308065
C	C23	3.455772	7.032880	12.896692
O	O2	3.883027	9.543283	13.390911
C	C6	-0.872613	6.155451	14.997180
H	H6	-1.023061	5.389331	15.502454
C	C3	-0.452770	8.466186	13.525209
H	H3	-0.325578	9.246090	13.034833
C	C5	-1.875286	6.663017	14.207754
H	H5	-2.698583	6.232479	14.166355
O	O3	1.977234	8.924847	11.951884
C	C9	3.764799	6.472087	16.567047
H	H9	3.715208	5.550728	16.682897
C	C4	-1.660683	7.804849	13.481175
H	H4	-2.344381	8.140278	12.947369
C	C19	0.747042	9.558309	18.961351
H	H19	0.602386	9.220951	19.815840
C	C21	0.473581	11.305825	17.356363
H	H21	0.148669	12.148203	17.133339
C	C22	1.164967	10.555690	16.427606
H	H22	1.298811	10.894671	15.572001
C	C11	5.050061	7.077699	16.928121
C	C20	0.270871	10.792883	18.615247
C	C12	6.217676	6.356694	16.669738
H	H12	6.162603	5.525151	16.256979
C	C16	5.149480	8.310070	17.557646
H	H16	4.378718	8.798121	17.738467
C	C15	6.391382	8.823414	17.921392
H	H15	6.375233	9.664372	18.318174
C	C24	4.730913	6.750713	13.316130
H	H24	5.203242	7.484009	13.638790
C	C28	2.639658	6.040448	12.409463

H	H28	1.779324	6.235605	12.115117
C	C13	7.454514	6.864970	17.020545
H	H13	8.248517	6.416031	16.839578
C	C25	4.992913	5.298271	13.158351
H	H25	5.837170	5.007449	13.418474
Br	BR1	9.350161	8.499697	17.952322
C	C14	7.597034	7.843796	17.496902

Table S4. DFT / CAM-B3LYP / 6-311 ++ G (d,p) Coordinates of asymmetric unit D QBCP.

		Coordinates		
Atom	Label	x	y	z
C	C26	7.499022	-1.570748	14.765407
H	H26	7.256883	-2.466543	14.827798
C	C27	8.539663	-1.177380	15.086968
H	H27	9.043878	-1.898752	15.387592
S	S1	8.845499	2.739023	14.384841
Cl	CL1	12.277840	5.798578	7.656744
N	N1	9.831847	2.715257	13.046894
O	O1	10.244632	-0.897689	11.251587
C	C10	9.177780	2.591054	11.714712
H	H10	8.287832	2.997471	11.770916
C	C18	10.219322	2.898371	9.424691
H	H18	9.890987	2.060327	9.190630
C	C17	10.000102	3.380206	10.699259
C	C7	10.186513	0.263954	11.631164
C	C1	11.294935	0.864379	12.409463
C	C8	9.009097	1.125492	11.355906
C	C2	11.093859	2.035827	13.144515
C	C23	8.208220	1.112230	14.555889
O	O2	7.780965	3.622633	14.061669
C	C6	12.536605	0.234801	12.455401
H	H6	12.687053	-0.531319	11.950127
C	C3	12.116762	2.545536	13.927371
H	H3	11.989570	3.325440	14.417748
C	C5	13.539278	0.742367	13.244827
H	H5	14.362575	0.311829	13.286225
O	O3	9.686758	3.004197	15.500697
C	C9	7.899193	0.551437	10.885534
H	H9	7.948784	-0.369922	10.769684
C	C4	13.324675	1.884199	13.971405
H	H4	14.008373	2.219628	14.505212
C	C19	10.916950	3.637659	8.491230
H	H19	11.061606	3.300301	7.636741
C	C21	11.190411	5.385175	10.096217
H	H21	11.515323	6.227553	10.319242
C	C22	10.499025	4.635040	11.024975
H	H22	10.365181	4.974021	11.880580
C	C11	6.613931	1.157049	10.524459
C	C20	11.393121	4.872233	8.837333
C	C12	5.446316	0.436044	10.782843
H	H12	5.501389	-0.395499	11.195602
C	C16	6.514512	2.389420	9.894935
H	H16	7.285274	2.877471	9.714114
C	C15	5.272610	2.902764	9.531188
H	H15	5.288759	3.743722	9.134407
C	C24	6.933079	0.830063	14.136450
H	H24	6.460750	1.563359	13.813791
C	C28	9.024334	0.119798	15.043117

H	H28	9.884668	0.314955	15.337464
C	C13	4.209478	0.944320	10.432036
H	H13	3.415475	0.495381	10.613003
C	C25	6.671079	-0.622379	14.294229
H	H25	5.826822	-0.913201	14.034107
Br	BR1	2.313831	2.579047	9.500258
C	C14	4.066958	1.923146	9.955678

Table S5. DFT / CAM-B3LYP / 6-311 ++ G (d,p) charges ChelpG of asymmetric unit A QBCP

		Unit C	
**Label**	**Atom**	**Isolated**	**Embedded**
C26	**C**	-0.033813	-0.032238
H26	**H**	0.107402	0.10515
C27	**C**	0.00859	0.015539
H27	**H**	0.046906	0.050461
S1	**S**	1.211185	1.20862
CL1	**Cl**	-0.154639	-0.183823
N1	**N**	-0.541968	-0.55772
O1	**O**	-0.531583	-0.576697
C10	**C**	0.418622	0.553889
H10	**H**	0.019569	-0.047367
C18	**C**	-0.152862	-0.17318
H18	**H**	0.09943	0.153286
C17	**C**	0.006301	-0.023313
C7	**C**	0.584556	0.604549
C1	**C**	-0.258506	-0.246862
C8	**C**	-0.193979	-0.278361
C2	**C**	0.360587	0.331762
C23	**C**	-0.118941	-0.088825
O2	**O**	-0.583204	-0.612898
C6	**C**	-0.007289	-0.022094
H6	**H**	0.104395	0.130218
C3	**C**	-0.250174	-0.235405
H3	**H**	0.176343	0.186495
C5	**C**	-0.164473	-0.179525
H5	**H**	0.1203	0.132794
O3	**O**	-0.580642	-0.598071
C9	**C**	-0.044023	-0.012329
H9	**H**	0.061901	0.061358
C4	**C**	-0.003794	-0.013096
H4	**H**	0.094457	0.09743
C19	**C**	-0.063935	-0.050963
H19	**H**	0.108649	0.107361
C21	**C**	-0.032172	-0.057853
H21	**H**	0.105453	0.106844
C22	**C**	-0.213931	-0.190932
H22	**H**	0.141631	0.132254
C11	**C**	0.12697	0.14619
C20	**C**	0.077901	0.099572
C12	**C**	-0.199912	-0.226716
H12	**H**	0.14486	0.156823

C16	**C**	-0.167947	-0.178597
H16	**H**	0.107597	0.137139
C15	**C**	-0.098218	-0.047682
H15	**H**	0.117348	0.111599
C24	**C**	-0.06471	-0.048405
H24	**H**	0.08527	0.049035
C28	**C**	-0.083637	-0.124861
H28	**H**	0.081566	0.092318
C13	**C**	0.077816	0.111957
H13	**H**	0.047043	0.048659
C25	**C**	-0.08953	-0.085927
H25	**H**	0.068175	0.109722
BR1	**Br**	-0.132929	-0.145865
C14	**C**	0.055989	-0.001419

Table S6. DFT / CAM-B3LYP / 6-311 ++ G (d,p) charges ChelpG of asymmetric unit B QBCP.

Label	Atom	Unit B	
		**Isolated**	**Embedded**
C26	**C**	-0.033813	-0.032209
H26	**H**	0.107402	0.105151
C27	**C**	0.00859	0.015461
H27	**H**	0.046905	0.050433
S1	**S**	1.211185	1.208649
CL1	**Cl**	-0.154639	-0.183784
N1	**N**	-0.541968	-0.557693
O1	**O**	-0.531583	-0.576719
C10	**C**	0.418622	0.554083
H10	**H**	0.019569	-0.047502
C18	**C**	-0.152862	-0.17319
H18	**H**	0.09943	0.153306
C17	**C**	0.006301	-0.02336
C7	**C**	0.584556	0.604575
C1	**C**	-0.258506	-0.246865
C8	**C**	-0.193978	-0.278485
C2	**C**	0.360587	0.331768
C23	**C**	-0.118941	-0.088887
O2	**O**	-0.583204	-0.612884
C6	**C**	-0.007288	-0.022106
H6	**H**	0.104394	0.130199
C3	**C**	-0.250174	-0.235336
H3	**H**	0.176343	0.186504
C5	**C**	-0.164473	-0.179566
H5	**H**	0.120301	0.132759
O3	**O**	-0.580641	-0.5981
C9	**C**	-0.044023	-0.012256
H9	**H**	0.061901	0.061395
C4	**C**	-0.003793	-0.013109
H4	**H**	0.094457	0.097423
C19	**C**	-0.063935	-0.050948
H19	**H**	0.108649	0.10736
C21	**C**	-0.032172	-0.057823
H21	**H**	0.105453	0.106856
C22	**C**	-0.213931	-0.190933
H22	**H**	0.141631	0.13225
C11	**C**	0.126972	0.146168
C20	**C**	0.0779	0.099545
C12	**C**	-0.199913	-0.226721

H12	**H**	0.14486	0.156843
C16	**C**	-0.167948	-0.178522
H16	**H**	0.107597	0.137113
C15	**C**	-0.098218	-0.047692
H15	**H**	0.117348	0.111565
C24	**C**	-0.064711	-0.048329
H24	**H**	0.08527	0.048981
C28	**C**	-0.083638	-0.124761
H28	**H**	0.081566	0.092196
C13	**C**	0.077817	0.111997
H13	**H**	0.047042	0.048709
C25	**C**	-0.089529	-0.085949
H25	**H**	0.068175	0.109745
BR1	**Br**	-0.132929	-0.145872
C14	**C**	0.055988	-0.001433

Table S7. DFT / CAM-B3LYP / 6-311 ++ G (d,p) charges ChelpG of asymmetric unit C QBCP.

Label	Atom	Unit C	
		**Isolated**	**Embedded**
C26	**C**	-0.033812	-0.032238
H26	**H**	0.107402	0.10515
C27	**C**	0.008589	0.01554
H27	**H**	0.046906	0.050461
S1	**S**	1.211184	1.208619
CL1	**Cl**	-0.154639	-0.183823
N1	**N**	-0.541967	-0.557719
O1	**O**	-0.531583	-0.576697
C10	**C**	0.41862	0.553888
H10	**H**	0.01957	-0.047367
C18	**C**	-0.152863	-0.173181
H18	**H**	0.09943	0.153286
C17	**C**	0.006303	-0.023311
C7	**C**	0.584555	0.604548
C1	**C**	-0.258506	-0.246862
C8	**C**	-0.193978	-0.278361
C2	**C**	0.360587	0.331762
C23	**C**	-0.118941	-0.088824
O2	**O**	-0.583203	-0.612898
C6	**C**	-0.007288	-0.022093
H6	**H**	0.104394	0.130218
C3	**C**	-0.250174	-0.235405
H3	**H**	0.176343	0.186496
C5	**C**	-0.164473	-0.179526
H5	**H**	0.1203	0.132794
O3	**O**	-0.580642	-0.59807
C9	**C**	-0.044024	-0.012329
H9	**H**	0.061902	0.061358
C4	**C**	-0.003794	-0.013096
H4	**H**	0.094457	0.09743
C19	**C**	-0.063934	-0.050962
H19	**H**	0.108649	0.107361
C21	**C**	-0.032171	-0.057852
H21	**H**	0.105452	0.106844
C22	**C**	-0.213933	-0.190934
H22	**H**	0.141632	0.132255
C11	**C**	0.126973	0.14619
C20	**C**	0.0779	0.099572
C12	**C**	-0.199914	-0.226716

H12	**H**	0.144861	0.156823
C16	**C**	-0.167949	-0.178596
H16	**H**	0.107597	0.137138
C15	**C**	-0.098217	-0.047683
H15	**H**	0.117347	0.111599
C24	**C**	-0.06471	-0.048405
H24	**H**	0.08527	0.049035
C28	**C**	-0.083638	-0.124863
H28	**H**	0.081566	0.092318
C13	**C**	0.077818	0.111956
H13	**H**	0.047042	0.04866
C25	**C**	-0.08953	-0.085926
H25	**H**	0.068175	0.109722
BR1	**Br**	-0.132929	-0.145866
C14	**C**	0.055988	-0.001418

Table S8. DFT / CAM-B3LYP / 6-311 ++ G (d,p) charges ChelpG of asymmetric unit D QBCP.

Label	Atom	Unit D	
		**Isolated**	**Embedded**
C26	**C**	-0.033813	-0.032209
H26	**H**	0.107402	0.105151
C27	**C**	0.00859	0.015461
H27	**H**	0.046905	0.050433
S1	**S**	1.211184	1.208648
CL1	**Cl**	-0.154639	-0.183783
N1	**N**	-0.541967	-0.557692
O1	**O**	-0.531583	-0.576719
C10	**C**	0.41862	0.55408
H10	**H**	0.01957	-0.047502
C18	**C**	-0.152863	-0.173191
H18	**H**	0.09943	0.153306
C17	**C**	0.006304	-0.023357
C7	**C**	0.584556	0.604576
C1	**C**	-0.258507	-0.246866
C8	**C**	-0.193978	-0.278484
C2	**C**	0.360587	0.331768
C23	**C**	-0.118941	-0.088887
O2	**O**	-0.583203	-0.612883
C6	**C**	-0.007288	-0.022105
H6	**H**	0.104394	0.130199
C3	**C**	-0.250174	-0.235335
H3	**H**	0.176343	0.186504
C5	**C**	-0.164473	-0.179565
H5	**H**	0.1203	0.132758
O3	**O**	-0.580642	-0.5981
C9	**C**	-0.044023	-0.012256
H9	**H**	0.061901	0.061395
C4	**C**	-0.003794	-0.01311
H4	**H**	0.094457	0.097424
C19	**C**	-0.063933	-0.050946
H19	**H**	0.108648	0.107359
C21	**C**	-0.032171	-0.057821
H21	**H**	0.105452	0.106856
C22	**C**	-0.213934	-0.190936
H22	**H**	0.141632	0.132251
C11	**C**	0.126972	0.146167
C20	**C**	0.077899	0.099543
C12	**C**	-0.199914	-0.226721
H12	**H**	0.14486	0.156843

C16	**C**	-0.167948	-0.178521
H16	**H**	0.107597	0.137113
C15	**C**	-0.098217	-0.047692
H15	**H**	0.117348	0.111565
C24	**C**	-0.064711	-0.048329
H24	**H**	0.08527	0.048981
C28	**C**	-0.083638	-0.124761
H28	**H**	0.081566	0.092195
C13	**C**	0.077818	0.111996
H13	**H**	0.047042	0.048709
C25	**C**	-0.089529	-0.085949
H25	**H**	0.068175	0.109745
BR1	**Br**	-0.132929	-0.145873
C14	**C**	0.055988	-0.001432

Table S9. DFT / CAM-B3LYP / 6-311 ++ G (d,p) Dipole moment of asymmetric unit A QBCP

**Step**	**Dipole Moment**
0	3.9269
1	4.7831
2	4.9763
3	5.0302
4	5.0421
5	5.0459
6	5.0468
7	5.0472
8	5.0472
9	5.0473
10	5.0473
11	5.0473
12	5.0473
13	5.0473
14	5.0473
15	5.0473
16	5.0473
17	5.0473
18	5.0473
19	5.0473
20	5.0473

Table S10. DFT / CAM-B3LYP / 6-311 ++ G (d,p) Dipole moment of asymmetric unit B QBCP

**Step**	**Dipole Moment**
0	3.9269
1	4.782
2	4.9748
3	5.0286
4	5.0404
5	5.0443
6	5.0451
7	5.0454
8	5.0455
9	5.0456
10	5.0456
11	5.0456
12	5.0456
13	5.0456
14	5.0456
15	5.0456
16	5.0456
17	5.0456
18	5.0456
19	5.0456
20	5.0456

Table S11. DFT / CAM-B3LYP / 6-311 ++ G (d,p) Dipole moment of asymmetric unit C QBCP

**Step**	**Dipole Moment**
0	3.9269
1	4.7831
2	4.9763
3	5.0302
4	5.0421
5	5.0459
6	5.0468
7	5.0472
8	5.0472
9	5.0473
10	5.0473
11	5.0473
12	5.0473
13	5.0473
14	5.0473
15	5.0473
16	5.0473
17	5.0473
18	5.0473
19	5.0473
20	5.0473

Table S12. DFT / CAM-B3LYP / 6-311 ++ G (d,p) Dipole moment of asymmetric unit D QBCP

**Step**	**Dipole Moment**
0	3.9269
1	4.782
2	4.9748
3	5.0286
4	5.0404
5	5.0443
6	5.0451
7	5.0454
8	5.0455
9	5.0456
10	5.0456
11	5.0456
12	5.0456
13	5.0456
14	5.0456
15	5.0456
16	5.0456
17	5.0456
18	5.0456
19	5.0456
20	5.0456

Table S13. DFT / CAM-B3LYP / 6-311 ++ G (d,p) *IDRI* Average Second Hyperpolarizability *(esu)*

IDRI* Average Second Hyperpolarizability *(esu)		
	Unit A	
Gamma	**Isolated**	**Embedded**
**|| (0;0.0.0)**	6.62678E-35	6.70564E-35
**|| (-w;w.0.0) w= 1906.4nm**	7.11892E-35	7.22992E-35
**|| (-w;w.0.0) w= 1064.0nm**	8.34326E-35	8.54306E-35
**|| (-w;w.0.0) w= 532.0nm**	1.74796E-34	1.87154E-34

Table S14. DFT / CAM-B3LYP / 6-311 ++ G (d,p) average linear polarizability

**Linear Polarizability *(esu)***		
	Unit A	
Alpha	**Isolated**	**Embedded**
**<α> (0;0)**	5.32776E-23	5.33605E-23
**<α> (-w;w) w= 1906.4nm**	5.35542E-23	5.36412E-23
**<α> (-w;w) w= 1064.0nm**	5.4191E-23	5.42883E-23
**<α> (-w;w) w= 532.0nm**	5.75016E-23	5.76748E-23

Table S15. DFT / CAM-B3LYP / 6-311 ++ G (d,p) the dynamic third-order nonlinear susceptibility

X(3) (-w; w. w. -w) **The Dynamic Third-Order Nonlinear Susceptibility** ***(S.I) m²/V²***		
	**Isolated**	**Embedded**
**X(3)** **<γ (-w; w. w. -w)> ; w = (o.o.o.o)**	8.23533E-21	8.3615E-21
**X(3)** **<γ (-w; w. w. -w)> ; w = 1906.4nm**	8.94719E-21	9.11902E-21
**X(3)** **<γ (-w; w. w. -w)> ; w = 1064.0nm**	1.07629E-20	1.10648E-20
**X(3)** **<γ (-w; w. w. -w)> ; w = 532.0nm**	2.58936E-20	2.79292E-20

Table S16. DFT / CAM-B3LYP / 6-311 ++ G (d,p) the refractive index. Clausius-Mossotti Relation

n **- Refractive Index** ***Clausius-Mossotti Relation***		
	**Isolated**	**Embedded**
**$n_{<\alpha>}$ (0;0)**	1.620376849	1.621580878
**$n_{<\alpha>}$ (-w;w) w= 1906.4nm**	1.624398594	1.625666197
**$n_{<\alpha>}$ (-w;w) w= 1064.0nm**	1.633706209	1.635134391
**$n_{<\alpha>}$ (-w;w) w= 532.0nm**	1.683230353	1.685875792

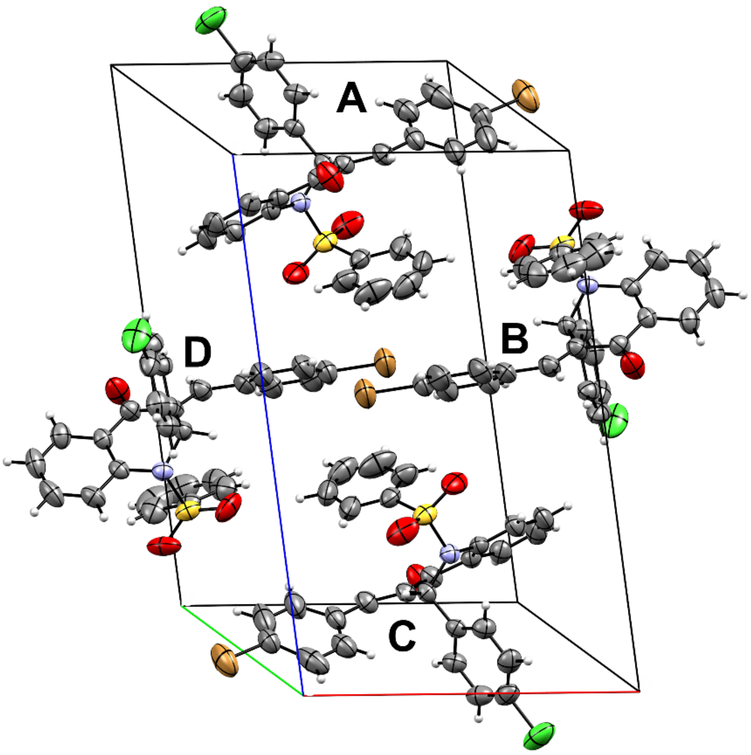

Figure S1. The unit cell with the four asymmetric units A.B.C. and D.

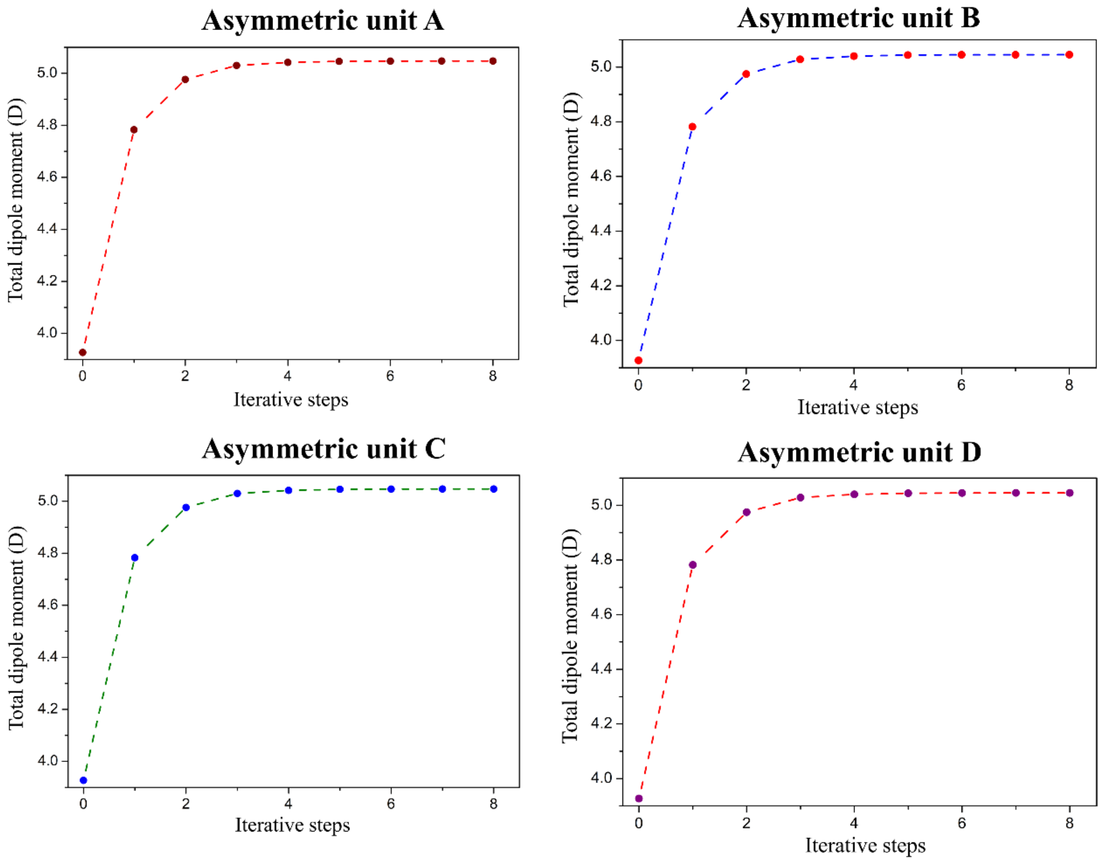

Figure S2. Dipole Moment of the four asymmetric units A.B.C. and D.

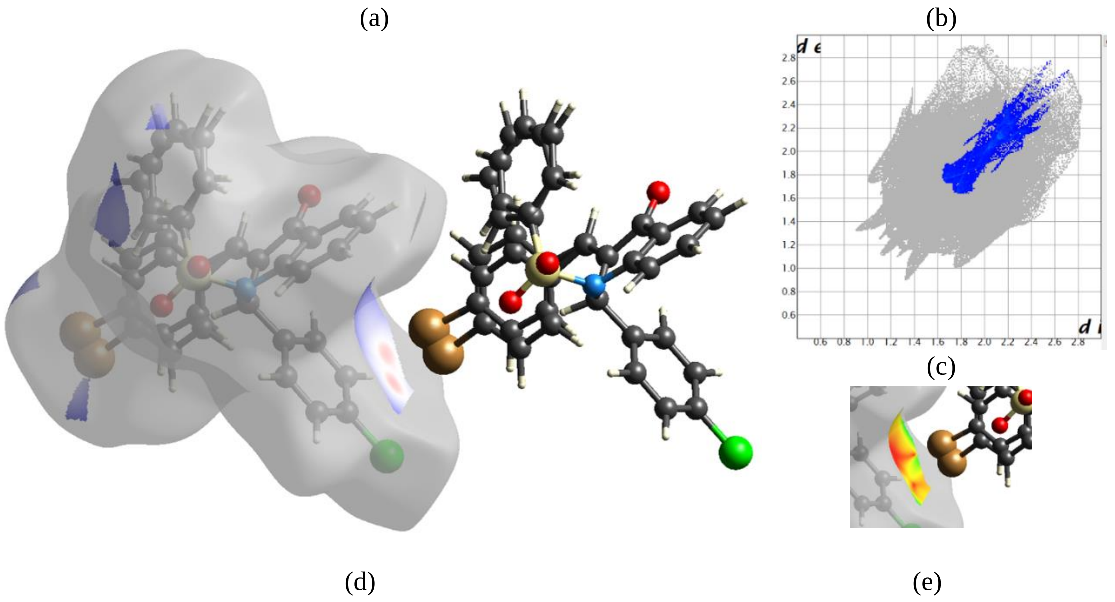

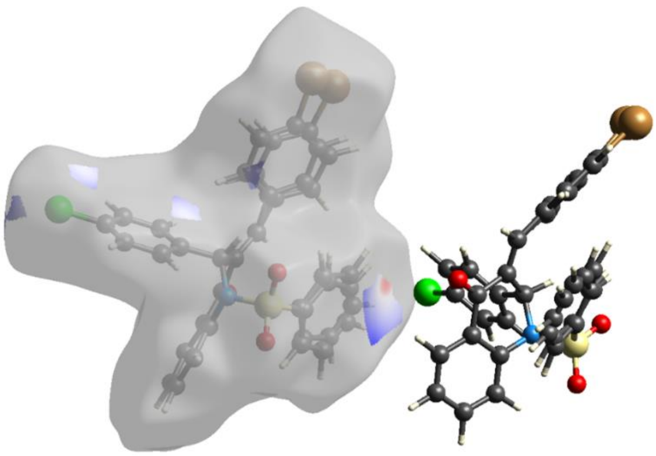

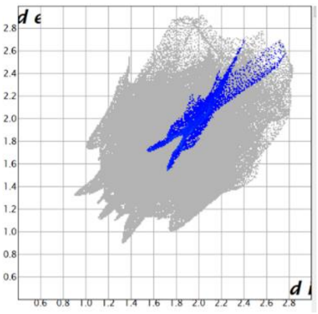

(f)

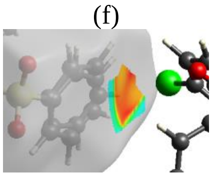

(g)

(h)

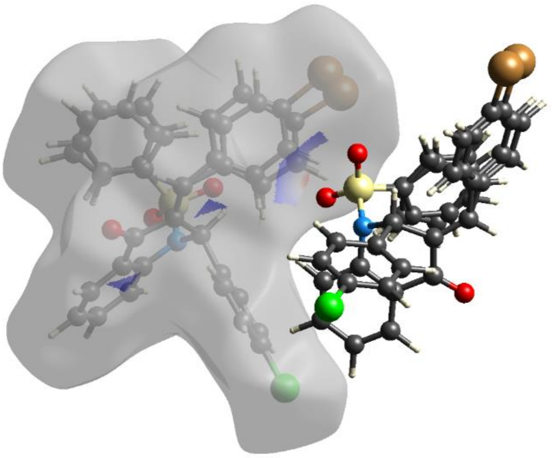

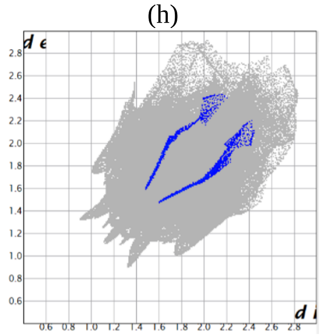

(i)

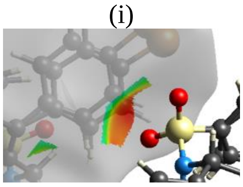

Figure S3. The d_norm property of Hirshfeld surfaces interactions presenting red spots between atoms. Fingerprints show the frequency of the type of interactions in the structure. The shape index shows by the red concave regions, a characteristic of aromatic ring involved in atom···π interactions. In d_norm property, the presence of red spots in surface between two atoms indicates an interaction, as seen in (a), (d) and (g). The shape index indicates the localization of hollows and bumps in surface and allows the identification of atom···π interactions, where the presence of a hollow (concave, red) region appears in the surface between an external donor atom and the aromatic ring (Figures (c), (f) and (i)).). The intermolecular interaction frequency of each combination of elements from inside and outside surface is mapped in fingerprint 2D plot ((b), (e) and (h)).

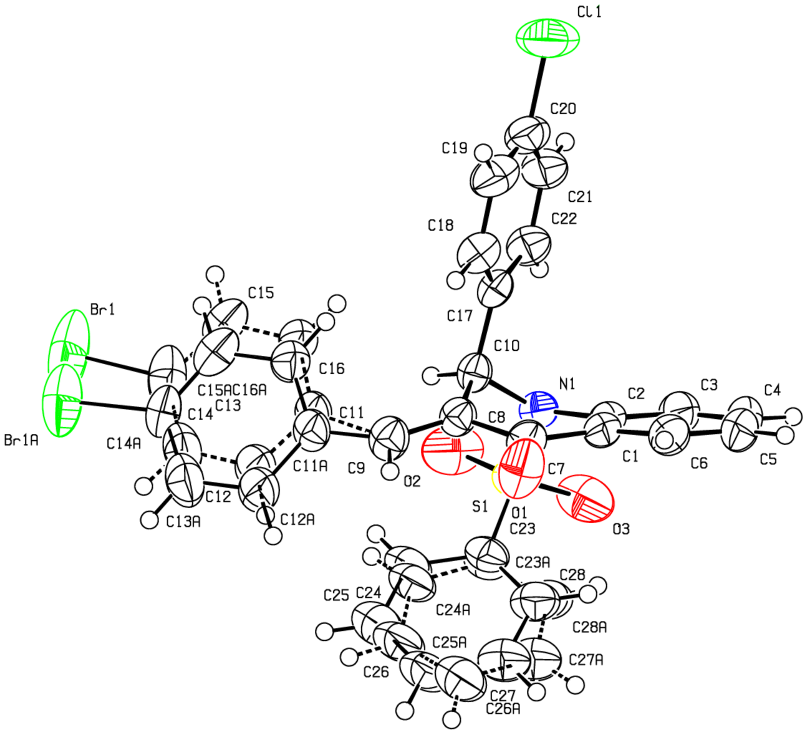

Figure S4. ORTEP map of the QBCP asymmetric unit with 50% probability. Disordered atoms have a suffix A.
